# Analysis of Tuberculosis Preventive Treatment Cascade Among People With Human Immunodeficiency Virus in Georgia: A Mixed-Methods Study

**DOI:** 10.1093/ofid/ofaf768

**Published:** 2025-12-15

**Authors:** Mariana Buziashvili, Davit Baliashvili, Akaki Abutidze, Nikoloz Chkhartishvili, Nestani Tukvadze, Otar Chokoshvili, Jack DeHovitz, Mamuka Djibuti

**Affiliations:** Scientific Research Unit, National Center for Tuberculosis and Lung Diseases, Tbilisi, Georgia; Department of Medicine, Tbilisi State University, Tbilisi, Georgia; Partnership for Research and Action for Health, Tbilisi, Georgia; T. Tsertsvadze Infectious Diseases, AIDS and Clinical Immunology Research Center, Tbilisi, Georgia; T. Tsertsvadze Infectious Diseases, AIDS and Clinical Immunology Research Center, Tbilisi, Georgia; Scientific Research Unit, National Center for Tuberculosis and Lung Diseases, Tbilisi, Georgia; Department of Medicine, Swiss Tropical and Public Health Institute, Allschwil, Switzerland; T. Tsertsvadze Infectious Diseases, AIDS and Clinical Immunology Research Center, Tbilisi, Georgia; Department of Medicine, State University of New York Downstate Health Sciences University, Brooklyn, New York, USA; Partnership for Research and Action for Health, Tbilisi, Georgia

**Keywords:** barriers and facilitators to TPT implementation, care cascade, HIV, people with HIV, tuberculosis preventive treatment

## Abstract

**Background:**

Tuberculosis preventive treatment (TPT) is crucial for reducing tuberculosis (TB) incidence and related mortality among people with human immunodeficiency virus (HIV); however, its implementation in Georgia faces challenges. In this study, we aimed to explore the TPT care cascade among people with HIV (PWH) in Georgia.

**Methods:**

Using a mixed-methods approach, we assessed TPT uptake, adherence, and impact on TB development within the 2019–2020 cohort of newly diagnosed PWH across 4 major HIV service providers in Georgia. With qualitative analysis under the Consolidated Framework for Implementation Research, we identified barriers and facilitators to its implementation.

**Results:**

Among 1165 PWH, only 11.8% initiated TPT with isoniazid. Thirty-two developed active TB (incidence rate, 10/1000 person-years [95% confidence interval, 9.6–10.4]), none of whom received TPT. Only 43% of 137 PWH on TPT adhered for 3–6 months; 29 (21.1%) completed the full course. The study revealed poor TPT service coordination, worsened by major data limitations. Interviews identified several barriers to effective TPT implementation, summarized into 3 broad categories: the need for TPT service integration into HIV care, the potential development of an integrated electronic data system, and training gaps.

**Conclusions:**

Our study revealed low TPT coverage among Georgian PWH and significant data gaps. Findings underscore the need to reevaluate the TPT care cascade, emphasizing improved record-keeping and reporting practices through an integrated electronic system. Enhancing access by integrating TPT into HIV care, reducing stigma through streamlined referrals, and strengthening healthcare worker training are critical to increasing TPT uptake and ultimately reducing TB morbidity and mortality among PWH in Georgia.

## Teaser

Low tuberculosis preventive treatment (TPT) uptake and poor adherence among people with HIV in Georgia highlight critical gaps in service coordination, data systems, and training. Integration of TPT into HIV care and electronic record improvements are key to reducing tuberculosis burden.

Tuberculosis (TB) remains a major global health threat, reclaiming its position as the leading cause of death from a single infectious agent, with an estimated one-quarter of the world's population being infected with *Mycobacterium tuberculosis* [1, 2]. About 5%–10% of those infected develop active TB, mostly within the first 2–5 years following the initial infection [3, 4]. Human immunodeficiency virus (HIV) significantly increases the risk of developing active TB, making TB a leading cause of morbidity and mortality among people with HIV (PWH) globally [5, 6]. In 2023, an estimated 10.8 million people developed TB, with an estimated 6% being among PWH [7]. During the same year, an estimated 1.1 million died of TB, including 167 000 PWH [2]. Delays and challenges in TB diagnosis and late or missed antiretroviral therapy (ART) initiation all contribute to preventable TB cases among PWH, significantly exacerbating their combined burden on public health [6–8].

Tuberculosis preventive treatment (TPT) is key to reducing TB-related incidence and mortality among PWH [9–11]. While global TPT uptake rose from 2.9 million in 2021 to 4.7 million in 2023, coverage among PWH declined from 2.2 million to 1.9 million [7]. The World Health Organization (WHO) recommends TPT for all PWH without active TB as part of a comprehensive care package [10]. Multiple studies have shown that alongside ART or alone, TPT lowers TB risk [12–15] and reduces mortality among PWH by 39% at 78 months after treatment initiation [16]. Nevertheless, despite a strong consensus on the importance of TB prevention in PWH, worldwide implementation appears heterogeneous and mainly restricted to better-resourced settings [17].

Until 2021, no explicit guidelines or precise control measures were implemented to monitor TPT provision in Georgia. Following the rollout of newly recommended TB infection treatment regimens and an enhanced TB preventive program [10], local National Tuberculosis Program (NTP) protocols were replaced with the first comprehensive guideline [18]. However, TPT delivery among Georgian PWH has been always governed by national HIV guidelines [19], delivered in collaboration with NTP. Despite active collaboration, the lack of formalized structure to monitor TPT uptake, adherence, and completion rates among PWH underscores a significant gap in our understanding of TPT implementation and effectiveness within the country.

In this article, we sought to explore TPT implementation landscape among newly diagnosed PWH during 2019–2020 in Georgia. The specific objectives were to (1) define countrywide coverage of the WHO-recommended 4-symptom screen (W4SS) for TB disease [20, 21], the number of TPT-eligible PWH, and the number recommended to and/or actually received TPT; (2) assess adherence among PWH initiating TPT; and (3) explore healthcare workers’ (HCW) perceived barriers and facilitators to implementing TPT among PWH in Georgia.

## METHODS

### Study Design

We employed a mixed-methods approach, including a retrospective review of 2019–2020 patient data from national HIV records and logs (including isoniazid [INH] prescription) to assess the TPT cascade in Georgia. Interviews with healthcare providers were conducted to explore perceived barriers and facilitators to TPT implementation among PWH.

### Study Settings

The country of Georgia provides universal access to free TB and HIV services through state programs and support from international donors, primarily the Global Fund to Fight AIDS, TB and Malaria. The study was conducted at 4 main HIV facilities in Georgia: the T. Tsertsvadze Infectious Diseases, AIDS and Clinical Immunology Research Center (IDACIRC) in Tbilisi and HIV facilities in 3 major cities (Kutaisi, Batumi, and Zugdidi, regional hubs offering comprehensive HIV services nationwide), minimizing the likelihood of missed diagnoses. The central TB facility in Tbilisi was included to interview HCWs also providing TPT for PWH, reflecting the current model where TPT services are predominantly managed by the NTP of Georgia, while 6- or 9-month INH regimens were provisioned through HIV care until late 2021.

The WHO recommends TB screening for all PWH using a standard clinical algorithm, including chest radiography and GeneXpert at baseline HIV evaluation. However, based on national HIV guidelines, only those with presumed TB or CD4 counts ≤200 cells/μL at baseline are referred to TB facilities for comprehensive examinations to rule out active TB and initiate TPT, while PWH with CD4 counts >200 cells/μL receive QuantiFERON testing, with TPT initiated only if results are positive [10, 19, 22].

### Study Population

For quantitative analysis, data were collected on all newly diagnosed PWH aged >18 years between January 2019 and December 2020 at the 4 main HIV facilities. For the qualitative component, 20 respondents, including HIV/TB clinicians, nurses, technical experts, and policymakers, were interviewed individually to capture diverse perspectives from persons directly or indirectly involved in TPT provision and ensure comprehensive understanding of TPT implementation landscape and the interconnected roles of HIV and TB services. The individual interviews were conducted during Fall 2024.

### Data Collection

Quantitative data of all PWH newly diagnosed during 2019–2020 were extracted from the de-identified IDACIRC database, with demographic, medical, and treatment details. TPT information was supplemented from paper-based logs, and the dataset was linked to the NTP surveillance database [23], using national IDs (subsequently removed to ensure data privacy) to evaluate TB disease development. TPT completion was defined as the documented receipt of the full 6-month regimen in clinic registries, reflecting programmatic completion, not adherence to daily medication intake, which was not part of the available data.

For the qualitative component, in-depth interviews were conducted in the Georgian language, using a Consolidated Framework of Implementation Research (CFIR)–informed semi-structured guide (see Supplementary Material 2, Interview Guide) to explore potential drivers of implementation success or failure [24]. All participants provided oral informed consent prior to the interview, which lasted 45–60 minutes either in-person or via video-conferencing platform. Interviews were audio-recorded, professionally transcribed, and the final transcripts were translated into English.

### Data Analysis

Univariate and bivariate statistical analyses described the study cohort and explored associations between key variables. Fisher exact test with mid-*P* value correction assessed the association between TPT receipt and active TB development within ART and non-ART groups. Interview transcripts were analyzed using a structured, CFIR-based approach, categorizing interview findings into the framework's 5 core domains, which enabled systematic identification of multilevel barriers and facilitators relevant to TPT delivery among PWH in Georgia. A coding template guided initial identification of themes around gaps in TPT delivery, which were further refined through dichotomous categorization as either barriers or facilitators. Identifiable details were removed to ensure anonymity. Illustrative quotes were selected to support each theme, with respondents categorized as “clinician” or “technical expert” to prevent identification within Georgia's small TB/HIV community.

### Ethics

Study approvals were obtained from the ethics committees of the National Center for Disease Control and Public Health of Georgia, the T. Tsertsvadze IDACIRC, and the National Center for Tuberculosis and Lung Diseases.

## RESULTS

To contextualize our findings, we refer to previously published quantitative analyses of the 2019–2020 PWH cohort in Georgia [23], which detailed insights into TB incidence and risk factors for active TB among PWH, as well as TPT initiation, establishing foundational data on the uptake and effectiveness of TPT. This study expands upon those findings by integrating additional qualitative insights, focusing on provider-identified barriers and facilitators to TPT implementation.

### Study Population Characteristics

Between 2019 and 2020, 1190 individuals were newly diagnosed with HIV in Georgia. The median age among the final study cohort of 1165 PWH (25 excluded due to missing ID) was 38 years (interquartile range [IQR], 30–48 years), 76.3% were male, and 72.3% were diagnosed in Tbilisi. Most transmissions were reported to be heterosexual (66.8%), while 14.8% were linked to injection drug use. At diagnosis, 28.9% of PWH had CD4 counts ≤200 μL (median, 328 [IQR, 159–509]). ART was initiated in 89.9% of cases, with a median time of 15 days (IQR, 10–29 days) postdiagnosis. After excluding those diagnosed within 2 months of HIV diagnosis, the TB incidence rate was 10 per 1000 person-years; mean time from HIV to TB diagnosis was 107.3 days (standard deviation, 424.1 days) [23] (Table 1).

**Table 1.** Cohort Baseline Characteristics and Tuberculosis Preventive Treatment Prevalence Among Newly Diagnosed People With HIV—Georgia, 2019–2020

Characteristic	No.	(%)^a^
Study cohort	1165^b^	(100)
Sex assigned at birth		
Female	276	(23.7)
Male	889	(76.3)
Age, y		
≤29	277	(23.8)
30–39	339	(29.1)
40–49	309	(26.5)
≥50	240	(20.6)
Mean (SD)	39.3	(11.9)
Median (IQR)	38	(30–48)
Mode of HIV transmission		
Heterosexual	778	(66.8)
Homosexual	199	(17.1)
IDU	172	(14.8)
Blood transfusion	4	(0.3)
Unknown	12	(1.0)
Initiated ART	1047	(89.9)
CD4 at baseline (performed within ∼2 weeks of diagnosis)^c^		
≤200 cells/μL	337	(29.0)
>200 cells/μL	746	(64.0)
Mean (SD)	357.2	(258.9)
Median (IQR)	328	(159–509)
HIV RNA at baseline, copies/mL^d^		
Mean (SD)	414 603	(125 679)
Median (IQR)	63 746	(12 130–264 000)
Started on TPT	137	(11.8)
Time to TPT initiation from HIV diagnosis, d		
Mean (SD)	43.2	(45.1)
Median (IQR)	27	(17–48)
TPT duration, mo		
<3	49	(35.8)
3–6	59	(43.1)
>6	29	(21.1)
Mean (SD)	3.8	(2.2)
Median (IQR)	3.9	(1.1–5.8)

Data are presented as No. (%) unless otherwise indicated.

Abbreviations: ART, antiretroviral therapy; HIV, human immunodeficiency virus; IDU, intravenous drug use; IQR, interquartile range; SD, standard deviation; TPT, tuberculosis preventive treatment.

^a^All percentages are calculated out of the total cohort included in the analysis (n = 1165 [100%]), except for the TPT duration variable, where the denominator reflects the number of individuals who initiated TPT (n = 137).

^b^Twenty-five of 1190 newly diagnosed PWH were excluded from study analysis due to inability to match across the HIV and TB databases.

^c^Missing: n = 82 (7%).

^d^Missing: n = 350 (30%).

### Quantitative Findings on the TPT Cascade

Of the newly diagnosed PWH, 137 (11.8%) initiated INH-based TPT, with a median start time of 27 days (IQR, 17–48 days) post–HIV diagnosis. TPT completion was a challenge: Only 29 (21.1%) completed the full 6-month course, while 49 (35.8%) discontinued treatment within 3 months (Table 1). Following local HIV guidelines, TPT uptake was higher among PWH with CD4 counts ≤200 cells/μL (n = 62/337 [18.4%]), compared to those with CD4 counts >200 cells/μL (n = 73/746 [9.8%]) (*P* < .001). TPT uptake did not differ significantly by year, with 12.9% of those diagnosed in 2019 (n = 84/651) versus 10.3% of those diagnosed in 2020 (n = 53/514) initiating TPT (*P* = .2).

Regardless of ART status, no PWH who received TPT developed active TB: Within the ART group, TB developed in 3% (n = 27) of the non-TPT subgroup versus none among TPT recipients (*P* = .02). In the non-ART group, TB occurred in 4.3% (n = 5) of the non-TPT subgroup, while no cases were observed among TPT recipients (*P* = .92) (Table 2). Given the presence of zero cells on our contingency tables, Fisher exact test with mid-*P* correction was used to assess statistical significance; however, we were unable to further calculate measures of association (eg, risk ratio, odds ratio), due to the inability to compute valid estimates in the presence of zero events.

**Table 2.** **Frequencies of Tuberculosis Disease Development Among People With HIV Receiving or Not Receiving Tuberculosis Preventive Treatment, Stratified by Antiretroviral Therapy Status** ^a^

Group	TPT Status	Developed Active TB, No. (%)	Did Not Develop Active TB, No. (%)	*P* Value^b^
ART group	Received TPT	0	135 (100^c^)	**.02**
	No TPT	27 (3)	885 (97)	
No ART group	Received TPT	0	2 (100)	.92
	No TPT	5 (4.3)	111 (95.7)	

The bolded *P* value indicates statistically significant association.

Abbreviations: ART, antiretroviral therapy; TB, tuberculosis; TPT, tuberculosis preventive treatment.

^a^n = 1165 (25 PWH excluded from frequency analysis due to inability to match across the HIV and TB databases).

^b^Due to the small sample size and the presence of low expected cell counts, the mid-*P* exact *P* value was reported since it offers a more accurate and less conservative alternative to Fisher exact test.

^c^The percentages reflect the proportion within each TPT exposure category (received TPT vs no TPT) stratified by ART status, ie, in each row, the denominator corresponds to the total number of individuals in that specific TPT group within the respective ART subgroup.

**Figure 1.**
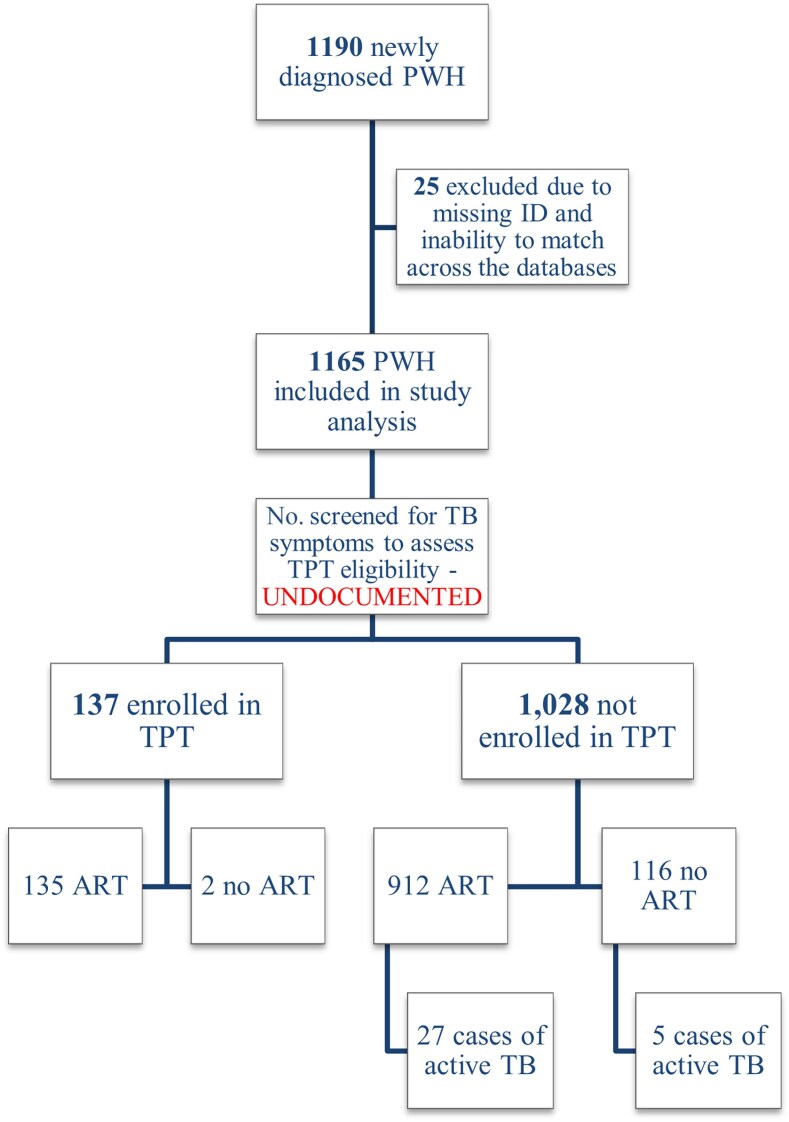
Flowchart of newly diagnosed people with HIV in Georgia during 2019–2020. Denominators decrease across steps of the flowchart and are based on the number of individuals at the immediately preceding step. Abbreviations: ART, antiretroviral therapy; PWH, people with human immunodeficiency virus; TB, tuberculosis; TPT, tuberculosis preventive treatment.

**Figure 2.**
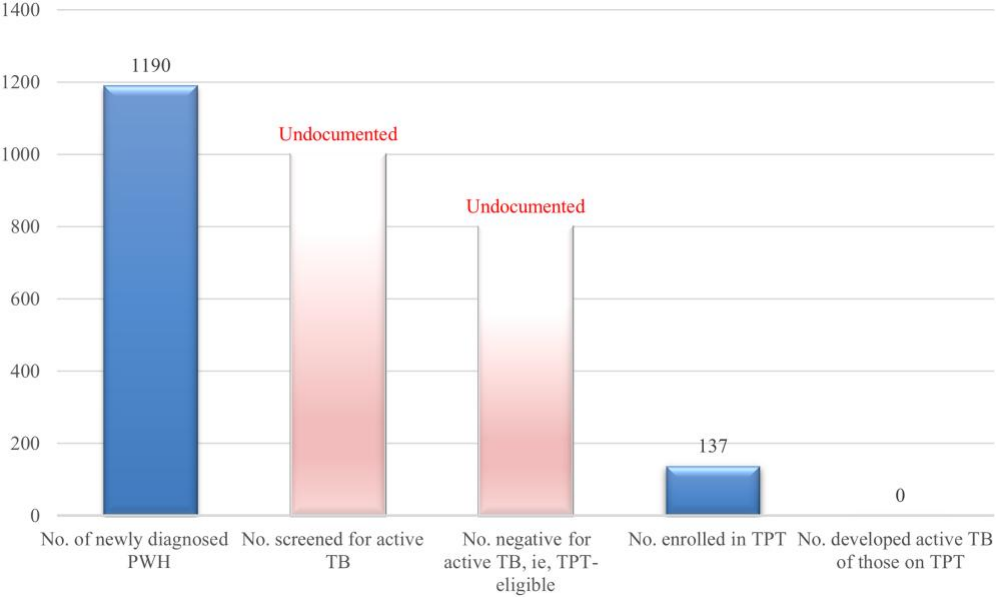
The TPT cascade of care among Georgian people with HIV, 2019–2020. Denominators decrease sequentially across steps and are based on the number of individuals at the immediately preceding step. However, due to the absence of data for 2 major steps of the cascade (number screened for TB disease and number eligible for TPT), specific denominators for these steps are unavailable. Therefore, the number enrolled in TPT is presented as a proportion of the total study cohort (N = 1190). Abbreviations: PWH, people with human immunodeficiency virus; TB, tuberculosis; TPT, tuberculosis preventive treatment.

Although Georgia's HIV care system follows robust protocols, including routine symptom screening at each visit and establishing TPT eligibility, certain elements pertaining to TPT care cascade were not fully documented in the available records (Figure 1). While physicians routinely screen for TB symptoms and document positive findings, detailed records regarding negative screening outcomes, formal TPT eligibility assessments, TPT initiation, adherence, and completion were not consistently captured in a standardized way, limiting comprehensive assessment of TPT cascade in our analysis (Figure 2).

### Qualitative Findings From the HCWs’ Interviews

Twenty healthcare providers (17 female, 3 men) were interviewed, including 15 clinicians (13 representing the HIV program, 2 representing the TB program), 1 nurse, 2 technical experts, and 2 policymakers from overall 6 facilities across Georgia. Interviews highlighted challenges to integrating TPT within the HIV care cascade, driven by systemic healthcare, sociopolitical, and organizational limitations, as well as limited stakeholder engagement. Key barriers and facilitators to effective TPT implementation were identified and analyzed across 5 CFIR domains (Supplementary Table 1): innovation characteristics, outer setting, inner setting, characteristics of individuals, and implementation process. Illustrative quotes from HCW interviews are listed in Table 3.

**Table 3.** Illustrative Quotes From Healthcare Workers’ In-Depth Interviews Mapped Across Consolidated Framework for Implementation Research Domains

CFIR Domain	Illustrative Quotes
I. Innovation characteristics	“TPT is essential and cost-effective; it's much less expensive than managing active TB. It's a critical preventive measure.” (R1, clinician, 53 y-o female) “The program's evidence-based approach and alignment with WHO recommendations make it highly credible and advantageous.” (R6, technical expert, 51 y-o female) “I think the program itself, including its design, is well constructed and is effective, however, the main drawback is that patients sometimes get lost between TB and HIV services. Integrating preventive treatment into HIV clinics would simplify access.” (R10, clinician, 43 y-o female)
II. Outer setting	“Patients in rural areas often tell us they can't travel to TB clinic because it's too far or expensive. Many avoid treatment altogether because they don't want others to know their HIV status.” (R1, clinician, 53 y-o female) “Localizing TPT within HIV clinics would solve so many problems. Patients wouldn’t need to travel or explain their situation to additional doctors.” (R7, clinician, 46 y-o male) “Stigma associated with attending TB facilities deters PWH from seeking TPT, as they fear being identified as HIV-positive.” (R20, technical expert, 57 y-o female) “DOT and VST are well justified. With limited mobility during COVID, 6-month INH was monitored via video, enabling the patient to adhere and complete the full course. Following the 3HP introduction in 2021, we applied the same approach.” (R6, technical expert, 51 y-o female) “3HP is conducted under video surveillance, making it easier for the patient to receive the service at home, creating a supportive environment.” (R19, technical expert, 57 y-o female) “Thanks to Global Fund, we’ve never had interruptions in drug supply or resources. This ensures the program runs smoothly.” (R12, technical expert, 39 y-o female) “Support from WHO and the Global Fund ensures consistent funding and medication supply, which are crucial for program sustainability.” (R3, clinician, 30 y-o male)
III. Inner setting	“While our institution is committed to TB prevention, the lack of integration between TB and HIV services creates logistical challenges for patients.” (R8, clinician, 54 y-o female) “There's a significant lack of skills—provider training on managing adverse events and new treatment protocols is insufficient, especially in regional clinics. Besides, TPT is still not considered as a priority for some clinicians and in my opinion, the lack of knowledge and evidence-based information is the reason for this.” (R6, technical expert, 51 y-o female) “We actively collaborate with TB specialists on a case-by-case basis, which strengthens our ability to address co-infections.” (R4, technical expert, 42 y-o male)
IV. Characteristics of individuals	“In our facility, it's the clinician's role to ensure eligibility for TPT, but the nurses often bridge the gap by educating patients and addressing their concerns.” (R2, clinician, 52 y-o female) “I would say the patient level is the most critical factor here. It's often very challenging to convince patients of the importance of initiating TB preventive treatment immediately, especially when they have no symptoms and feel fine. Most of them don’t perceive the urgency because they lack sufficient information.” (R7, clinician, 63 y-o female) “In my opinion, all of the levels involved have relevant competencies, but it's crucial their roles to be defined clearly. Everybody's role is to ensure that the policies are implemented effectively, but we rely on clinicians and nurses to carry out the daily work. Coordination between these roles is critical.” (R11, clinician, 53 y-o female) “Without support from higher levels, we wouldn’t be able to accomplish much. Even with the best intentions for patients, the lack of political will, adequate funding, and authority, limits access to services and our ability to strongly recommend TPT to patients. Without these structural supports, the relevant services simply wouldn’t exist.” (R17, clinician, 60 y-o female) “If all stakeholders, from policymakers to frontline providers, worked more cohesively, we could ensure better patient outcomes and make TPT more accessible.” (R13, clinician, 46 y-o male)
V. Process of implementation	“Ultimately, if we want TB chemoprophylaxis to be universally implemented for all HIV patients, we need three things: (1) a shared, integrated electronic database, (2) improved geographic accessibility, and (3) extensive training—not just in Tbilisi, but also in the regions—to ensure there is no information vacuum.” (R1, clinician, 53 y-o female) “Without an integrated electronic data system, it's difficult to track patients receiving TPT and coordinate care between TB and HIV services.” (R6, technical expert, 51 y-o female) “To improve [TPT] enrollment and ensure completion, I would recommend refining the existing mechanisms, for instance similarly to the existing VST approach, that tracks adherence through video and promptly flags missed doses, an integrated electronic system could significantly enhance patient tracking and allow timely interventions.” (R14, clinician, 48 y-o female) “I don’t recall much about the implementation process before; there were likely some training sessions and activities. However, when the treatment regimens changed—specifically with the introduction of rifapentine regimens, which I believe happened after the pandemic—we didn’t receive any training or preparatory activities.” (R9, clinician, 45 y-o female) “We need more training to confidently prescribe and manage TPT, especially in complex cases involving drug interactions.” (R11, clinician, 53 y-o female) “There is no structured framework for monitoring and evaluating the TPT program—assessments are only made during quarterly TB supervisions, and issues are addressed individually. To improve implementation, I would establish a unified group representing all key areas to ensure flexible and coordinated management.” (R6, technical expert, 51 y-o female)
Additional facilitators, highlighted during the interviews	“I believe the program's effectiveness could also improve significantly with the introduction of an incentive-based approach. For some, it could be a powerful motivator to initiate and complete preventive treatment. Same goes for the healthcare providers.” (R14, clinician, 48 y-o female) “Since patients undergoing TB treatment already receive financial vouchers, a similar incentive system could be introduced for TPT recipients. However, currently, there is no supporting evidence to guide the implementation of such a system.” (R19, technical expert, 57 y-o female) “Offering patients a financial voucher upon completing TPT could be highly effective. I recall an example during the COVID-19 vaccination campaign when many initially reluctant PWH, particularly elderly, participated in the campaign after incentive was allocated. Given that the country has only a few thousand PWH, implementing such approach for TPT would likely not pose a significant financial burden.” (R17, clinician, 60 y-o female)

Abbreviations: 3HP, 3-months of weekly isoniazid and rifapentine; CFIR, Consolidated Framework for Implementation Research; COVID-19, coronavirus disease 2019; DOT, directly observed treatment; HCW, healthcare worker; HIV, human immunodeficiency virus; INH, isoniazid; PWH, people with human immunodeficiency virus; TB, tuberculosis; TPT, tuberculosis preventive treatment; VST, video-supported treatment; WHO, World Health Organization; y-o, years-old.

#### Innovation Characteristics

The analysis of this domain identified a strong foundation for implementing TPT, with the majority of respondents emphasizing the national TB and HIV programs as key enablers. TPT was consistently perceived as highly beneficial and evidence-based, with many respondents agreeing that it is cost-effective, reduces mortality among PWH, and aligns well with international guidelines. This consensus highlights the program's relative advantage, particularly when compared to prior approaches in the country, as well as its compatibility within the existing healthcare framework. However, several respondents also noted challenges related to the complexity of integrating TPT services into existing HIV care, underscoring the need for streamlined approaches to enhance service delivery.

#### Outer Setting

The outer setting analysis revealed key barriers and facilitators to TPT implementation, with barriers predominantly manifested through geographical accessibility challenges and concerns about HIV status disclosure, while external influences and support were emphasized as strong facilitators. The respondents frequently highlighted that patients, particularly those in remote or rural areas, face difficulties traveling to regional TB clinics for monthly TPT doses and tests, which not only discourages patients from seeking TPT but also delays its initiation, leading to health risks and reduced program effectiveness in reaching all eligible PWH. Anticipated HIV stigma further deters care, with PWH avoiding nearby TB facilities to protect confidentiality. A decentralized model—integrating TPT with HIV services—was widely recommended to reduce logistical and stigma-related challenges.

Conversely, facilitators included innovations like video-supported treatment, which improves adherence and convenience, with around a quarter of respondents highlighting this as impactful. Strong support from international organizations, particularly the Global Fund and WHO, was also noted for ensuring drug supply, updating guidelines, and enabling effective implementation of national TPT policies.

#### Inner Setting

Strong institutional commitment to TB prevention was recognized by all respondents, with most emphasizing the importance of integrating TPT services to improve patient outcomes. While many institutions viewed TPT as essential, some providers still undervalued its importance and lacked necessary skills for effective delivery. Despite existing collaboration between HIV and TB programs, several respondents identified the integration of TPT services as an ongoing challenge, especially in regional areas. Moreover, the reliance on informal communication channels between the 2 programs contributes to inefficiencies and missed opportunities for comprehensive care. Formalizing these systems via integration of TPT services into HIV care and improving cross-program coordination were widely recommended to enhance service delivery and outcomes.

#### Characteristics of Individuals

This domain highlighted individual-level factors influencing TPT implementation, emphasizing the roles, competencies, and attitudes of different stakeholders from decision-makers and executive leaders to implementors and deliverers. Respondents identified both barriers and facilitators tied to these individual-level characteristics. Most agreed that successful TPT implementation heavily relies on the effective collaboration across stakeholder levels. Clinicians were central to identifying eligible patients and initiating treatment, while nurses supported patient education and adherence. Program coordinators and technical experts were seen as instrumental in aligning TPT with broader HIV/TB program objectives, ensuring effective policy implementation and resource allocation. Although all the respondents agreed that none of this would have been possible without effective leadership and guidance from the decision-making level. Improved communication, coordination, and shared accountability among all stakeholders were identified as critical for further strengthening TPT delivery.

#### Process of Implementation

Barriers to implementation process included insufficient capacity building, poor data recording practices, and weak monitoring and evaluation mechanisms. Most respondents recommended developing a structured monitoring framework to systematically assess progress and address barriers. The absence of an integrated electronic health record (EHR) system was frequently noted, limiting data sharing, adherence tracking, and outcome monitoring, ultimately affecting TPT effectiveness. Additional financial and human resources were seen as crucial for formalizing interinstitutional coordination between HIV and TB programs.

All respondents agreed that the program effectiveness heavily depends on healthcare providers’ knowledge and skills, with gaps in regular training and TPT-focused updates seen as significant obstacles. While prior intensive training was reported, many emphasized the need for regular updates and refresher courses, particularly with the introduction of new regimens and guidelines. The lack of recent training and insufficient focus on TPT-specific practices during workshops were seen as significant gaps affecting the quality of care. Regular evaluation of implementation was also highlighted by many to improve TPT delivery. In contrast, strong provider commitment and recognition of TPT's benefits were mentioned as significant facilitators to TPT implementation.

Many clinicians strongly supported using incentive-based approaches to enhance TPT implementation, citing their past success with other healthcare interventions. They noted that incentives could motivate both providers and TPT recipients, fostering greater engagement and adherence, helping providers prioritize TPT delivery, and easing patient burdens like travel costs and lost income. This dual-targeted approach was seen as a practical way to boost engagement, adherence, and overall program effectiveness.

## DISCUSSION

The study provides unique insights into the TPT care cascade among PWH in Georgia, revealing significant implementation gaps despite the universal access to TB/HIV services, existence of comprehensive guidelines, and active personal collaboration between the 2 programs. The lack of standardized approaches for TPT provision among PWH and inconsistent data documentation practices have resulted in substantial information gaps and discrepancies, undermining effective program monitoring and evaluation.

We found that TPT coverage among newly diagnosed PWH in 2019–2020 was only 11.8%, with uptake being relatively higher (18.4%) among PWH presenting to HIV care with a CD4 count ≤200 cells/μL, which is a strict indication for TPT per local guidelines. The prevalence is substantially lower compared to that of the WHO European Region, where 55% of PWH newly enrolled in care initiated TPT only in 2019 (80% in 2020), as well as the neighboring country of Azerbaijan, where 66% of PWH on ART initiated TPT, and comparably Ukraine, with 30.9% of PWH completing INH-based TPT [25–28]. However, the prevalence was higher compared to Armenia, where only 2.7% of PWH on ART initiated TPT [25]. There is a noticeable paucity of research focusing on the uptake of TPT within the WHO European Region, or in countries that share sociodemographic, political, economic, and disease-burden characteristics similar to those of Georgia. Conversely, numerous studies from African and selected Asian countries have reported TPT uptake rates ranging from 14% to 87% [29–36]. Notably, a recent study in Uganda assessed the TPT uptake rate before and after the implementation of a targeted “100-day accelerated TPT intervention,” recording an increase of over 35% in TPT uptake following the campaign—from 25% to 61.2% [30]. These outcomes from a single targeted initiative suggest the potential for rapid improvement in TPT uptake rates among PWH in diverse settings. The efficacy of such targeted TPT interventions for reducing the incidence of active TB and related mortality among PWH has also been demonstrated in several systematic reviews and WHO guidelines [37–39]. However, implementation of these strategies encounters numerous challenges, including logistical issues, healthcare infrastructure deficits, socioeconomic barriers, and gaps in policy implementation, resulting in varying success across different regions and disparities in enactment of TB prevention policies [9, 37, 40–42]. Although the small sample size limits the generalizability of the findings in our case, results are consistent with the published research and support the conclusion that scaling up TPT could significantly reduce TB-related morbidity and mortality among PWH.

The lack of data at multiple levels of the TPT care cascade in Georgia significantly limited our ability to comprehensively assess the full spectrum of its implementation. Specifically, no data were available precisely on W4SS active TB screening among PWH (although standalone symptoms recorded without correlation to TB and/or any other comorbidities) or the number of TPT-eligible and TPT-recommended PWH. The inability to track each step of the cascade poses challenges not only for research but also for optimizing care strategies for PWH [36, 43, 44]. Programmatic changes in Georgia since the study period, including introduction of rifapentine-based regimens and broader access to interferon-γ release assay testing, may have improved TPT eligibility assessments and adherence. Hence, ongoing evaluation using updated data is required to assess the full impact of these changes in the years following this study. Of note, another limitation is that the study did not collect information on AIDS-defining and/or non-AIDS-related comorbidities, such as hepatitis coinfection, liver dysfunction, and mental health disorders, which may have influenced both clinical eligibility and provider decisions for initiating TPT. The absence of such variables limits our ability to assess whether differential TPT uptake was driven by underlying clinical concerns or programmatic gaps and may introduce bias into estimates of both uptake and outcomes.

The qualitative component of the study explored the reasons behind the existing gaps in TPT care cascade, providing context to support the interpretation of quantitative findings. The findings from HCW interviews underscore several critical barriers to TPT implementation in Georgia, including lack of service integration, HIV stigma, lack of data sharing, and insufficient provider training.

### Integration of TPT Services Into HIV Care

Geographical accessibility was a persistent barrier, with both distance and challenge of navigating referrals making it difficult to access TPT. For PWH, especially those in rural areas, seeking TB-specific services often meant disclosing HIV status to more people, increasing stigma and deterring care. Similar challenges have been noted in previous studies and underscore the importance of integrated service delivery [36, 41, 45]. As demonstrated in published studies, integrated care models can streamline treatment, reduce logistical and emotional burdens, and improve outcomes [41, 46, 47]. Addressing stigma—particularly around disclosure at TB clinics—remains essential for improving TPT uptake among PWH. This issue resonates with findings from literature, emphasizing the role of stigma in deterring patients from accessing care and highlighting the need for stigma-reduction strategies within TB and HIV programs [46, 48].

### Integrated Electronic Data System

A major barrier to effective TPT implementation is the lack of an integrated data sharing platform between the programs. This gap prevents tracking of the full TPT cascade, impeding both research efforts and care strategy optimization for PWH, as well as contributing to fragmented care and logistical challenges. Studies show that an integrated electronic data system could improve communication, streamline workflows, and enable systematic tracking, ultimately strengthening TPT outcomes for PWH [49–52].

### Capacity Building

The training gap reflects a lack of regular updates and tailored capacity building for providers, especially in managing new TPT regimens and adverse events. This aligns with other studies, highlighting inadequate training as a key barrier to implementing evidence-based TB interventions in resource-constrained settings [31, 46]. Comprehensive and ongoing training across all aspects of TPT delivery is essential to strengthen the provider's capacity, ensure accurate record-keeping, and generate the evidence needed for effective program implementation [31].

In-depth interviews also highlighted several facilitators for TPT implementation, including the TB program's alignment with best practices and frequent guideline updates. External funding, especially from the Global Fund, was critical for resource availability, while innovations like video-supported treatment monitoring improved adherence [53]. Informal collaborative framework between HIV and TB specialists emerged as a major enabler, providing a strong foundation for addressing gaps and enhancing program reach [41, 54].

Our findings can be summarized into 2 key areas of recommendations for optimizing TPT among PWH in Georgia: first, actively integrating TPT services into HIV care to streamline approach, reduce logistical barriers, and address disclosure concerns; and second, establishing an integrated electronic data system to improve data sharing, track adherence, and strengthen monitoring and evaluation. Given the country's experience with digital health platforms, this recommendation is both feasible and timely. However, further evaluation of contextual barriers and facilitators to EHR implementation is needed to guide its design and sustainability. These steps, alongside sustained HCW capacity building and ongoing program evaluation, directly address key barriers and lay the groundwork for an effective, patient-centered TPT program.

## CONCLUSIONS

This study underscores the strengths and challenges of TPT implementation among PWH in Georgia. A strong evidence base, collaborative framework, and innovations like video-supported monitoring show promise for effective delivery. However, barriers such as stigma, geographic access issues, training gaps, limited service integration countrywide, and inconsistent data practices hinder success. Targeted strategies—particularly implementing an integrated EHR system—could improve data sharing and service coordination. A follow-up evaluation is recommended to assess the impact of rifapentine-based regimens and expanded latent TB infection testing on cascade performance.

## Supplementary Material

ofaf768_Supplementary_Data

## Supplementary Data

Supplementary materials are available at *Open Forum Infectious Diseases* online. Consisting of data provided by the authors to benefit the reader, the posted materials are not copyedited and are the sole responsibility of the authors, so questions or comments should be addressed to the corresponding author.
