# Supplementary material for: Analysis of Tuberculosis Preventive Treatment Cascade Among People With Human Immunodeficiency Virus in Georgia: A Mixed-Methods Study
Source: Open Forum Infect Dis. 2025 Dec 15;13(1):ofaf768. doi: 10.1093/ofid/ofaf768 (PMC12757687; doi:10.1093/ofid/ofaf768)
Supplement: ofaf768_Supplementary_Data [file ofaf768_supplementary_data.zip › SupplementaryMaterial2_interview_guide_MB.docx]

**A guide for in-depth interview among HIV/TPT service providers in Georgia**

*The interviewer explains the overall study objectives and procedures for participation, including providing information about the audio recording, and obtains written informed consent from the participant.*

**Introduction**

Thank you for agreeing to participate in this in-depth interview. You have been briefed about the study goal, alongside the guidelines and rights that come with your involvement in this research. Your written consent to participate has also been collected. Now, we are ready to begin.

You are informed that the study's focus is on assessing the context for implementing tuberculosis preventive treatment in HIV clinics, including TPT characteristics and related contextual factors at individual (provider), clinic (inner setting), and outer setting/system levels, and additionally, assessing the context for a potential implementation of an evidence-based practice for improving data recording and reporting on TPT in HIV clinics. As recognized in the preliminary results of our previous study looking at TB incidence rate among PWH, some major gaps were identified, specifically, within the recording and reporting of the majority of the clinical data, including baseline TB screening following the HIV diagnosis, TPT eligibility assessment, and TB preventive treatment initiation. Thus, the insights from these interviews will significantly contribute to the knowledge of TB preventive strategies, offering insights on existing barriers and facilitators to the operationalization of the TPT program in a real-world setting. The experiences and recommendations gained from the healthcare workers will aid in informing policymakers, program implementers, and help shaping future services and optimization of TB prevention and care among PWH.

I would like to remind you that our interview is expected to last for about 30-50 minutes. Some questions might seem sensitive or very personal to you. Please remember that you have the liberty to skip any question that you might find uncomfortable or to stop your participation at any moment. If you have any questions or need further explanation during the interview, feel free to ask. This interview will be audio-recorded for transcription and analysis. The recording will strictly be for our internal use, and your confidentiality will be fully protected. Please, if you have any questions or concerns about the process, share them with me now. Also, kindly reaffirm your consent to participate before we proceed.

| **Healthcare workers’ perceived barriers and facilitators to implementing TPT program** |
| --- |
| **Introductory Questions:** *[before going to the questions below, the interviewer will ask questions about the following socio-demographic information]*   - Age, Sex - Place of residence/work - Info about interviewee’s professional background, including their role and experience in providing HIV care? |
| **Domain – Innovation characteristics** *(evaluate the understanding of the TPT program, its’ source/evidence-base, design, complexity, and cost)* |
| 1. What do you think about the TPT program in general? Probe on the following:    - *Source (organization, that developed and/or visibly sponsored use of the innovation, is reputable, credible, and/or trustable)*    - *Evidence-base (has robust evidence supporting its effectiveness)*    - *Relative Advantage (is better than other available innovations or current practice)*    - *Complexity (is complicated, which may be reflected by its scope and/or the nature and number of connections and steps)*    - *Design (is well designed considering: patient-centered service delivery, human resource needs/ capacity, data system needs/capacity, the needed drugs/tests/consumables, governance)*    - *Cost (operating costs are affordable)* |
| **Domain – Outer setting** *(explore external influences on the implementation of TPT)* |
| 1. What do you think about external conditions for implementation of TPT program? Probe on the following:    - *Policies and laws (legislation, regulations, professional group guidelines and recommendations, or accreditation standards support implementation and/or delivery of the innovation)*    - *Local Conditions (economic, environmental, political, and/or technological conditions enable the MOH/ NCDC/ IDACIRC/ NCTLD to support implementation and/or delivery of the TPT)*    - *Financing (funding from external entities [e.g., GF] is available to implement and/or deliver the innovation)*    - *Partnerships & Connections (HIV clinics/ facilities are networked with external entities, including referral networks, academic affiliations, and professional organization networks)*    - *Critical Incidents (large-scale and/or unanticipated events disrupt implementation, e.g., COVID19, and/or delivery of the innovation)*    - *External Pressure (e.g., Performance Measurement Pressure for quality or benchmarking metrics or established service goals drive implementation and/or delivery of the innovation, e.g., from GF, WHO, MOH)* |
| **Domain – Inner setting** *(delve into the internal environment and its impact on TPT knowledge and implementation)* |
| 1. What do you think about the internal environment for implementation of TPT program? Specifically:    - *Structural characteristics (configuration of the inner environment and other tangible materials, including technological systems for communication, documentation, and data storage, management, reporting, organizational tasks and responsibilities within and between individuals and teams, that support functional performance of TPT)*    - *Communications (formal and informal relationships, networks, information sharing practices within and across the inner environment related to TPT program implementation and enhancement)*    - *Culture (shared values, beliefs, and norms around caring, supporting, and addressing the needs of TPT program recipients, i.e., PWH, deliverers, i.e., HCWs, and around the psychological safety, continual improvement, and using data to inform practice)*    - *Compatibility and relative priority (does the TPT fit within the current workflow, system, and process and is its’ delivery more important compared to other initiatives?)*    - *Incentive system (are there tangible and intangible incentives and rewards and/or disincentives and punishments supporting implementation and delivery of TPT?)*    - *Mission alignment (implementation and delivery of TPT is in line with the overarching goal of the HIV program)*    - *Resources (available resources to implement and deliver TPT: funding, human resources, supplies, guidance and/or training, etc.)* |
| **Domain – Characteristics of individuals** *(assess TPT-related individual beliefs, attitudes, and experiences)* |
| 1. Discuss the roles, competence, knowledge, skills, availability, and commitment of individuals with different levels of authority regarding TPT program implementation and delivery:    - *High-level leaders (decision-makers, executive leaders, directors)*    - *Mid-level leaders (leaders supervised by a high-level leader and who supervise others)*    - *Opinion leaders (individuals with informal influence on the attitudes and behaviors of others)*    - *Implementors (individuals with subject expertise, individuals leading implementation efforts, and other collaborators who support the TPT program implementation and delivery in practice)*    - *Deliverers and Recipients (individuals who directly or indirectly deliver or receive TPT program services)* |
| **Domain – Process of implementation** *(focus on the processes involved in implementing TPT guidelines and identifying knowledge gaps)* |
| 1. Describe the activities and strategies used to implement TPT at your clinic? Probe on the following:  - *Teaming (the degree to which individuals team up, coordinate and collaborate on tasks to support TPT implementation and delivery)* - *Needs assessment (priorities, preferences, and needs of people, i.e., TPT deliverers vs recipients)* - *Context assessment (collect information on barriers and facilitators to TPT implementation and delivery)* - *Planning (identification of roles and responsibilities, outlining specific steps and milestones, defining goals and measures for TPT implementation success)* - *Tailoring strategies (choosing and operationalization of implementation activities to address barriers, leverage facilitators and fit context)* - *Engaging (attract and encourage participation of TPT deliverers and recipients)* - *Doing (TPT implementation in small steps, tests, or cycles of change, to trial and cumulatively optimize delivery strategies)* - *Reflecting and Evaluating (collect and discuss quantitative and qualitative information about the success of TPT and its’ implementation)* - *Adapting (modify the TPT program strategies and/or inner environment for optimal fit and integration into work process)* |
| **Summary questions** *(This last section of our interview guide will close the interview and allow the participants to express their final thoughts about the topic)* |
| 1. What changes would you recommend improving the implementation of TPT program in Georgia? 2. Would you like to add/recommend anything related to TPT, that we have not covered during the interview? |

Now, we are concluding our interview. Thank you for your valuable participation. If you have anything else to add that we have not covered, please feel free to share. If you have any further questions or concerns, please contact me any time via email or phone.
