# Supplementary material for: Analysis of Tuberculosis Preventive Treatment Cascade Among People With Human Immunodeficiency Virus in Georgia: A Mixed-Methods Study
Source: Open Forum Infect Dis. 2025 Dec 15;13(1):ofaf768. doi: 10.1093/ofid/ofaf768 (PMC12757687; doi:10.1093/ofid/ofaf768)
Supplement: ofaf768_Supplementary_Data [file ofaf768_supplementary_data.zip › SupplementaryMaterial_Table1_TPTcarecascade_MBuziashvili.docx]

### Supplementary Table 1: Matrix summary of CFIR domains and constructs used for exploring the HCWs perspectives on TPT

| **CFIR Domains** | **Constructs** |
| --- | --- |
| I. Innovation Characteristics | Source (innovation/program coming from a credible source)  Evidence-base (has robust evidence supporting its effectiveness)  Relative Advantage (is better than other available innovations or current practice)  Complexity (is complicated, which may be reflected by its scope and/or the nature)  Design (patient-centered service delivery, human resource, data system, the needed consumables, governance)  Cost (operating costs are affordable) |
| II. Outer Setting | Policies and laws (legislation, regulations, professional)  Local Conditions (economic, environmental, political, and/or technological)  Financing (funding from external entities)  Partnerships & Connections (facilities are networked with external entities)  Critical Incidents (large-scale and/or unanticipated events disrupt implementation)  External Pressure (Performance Measurement Pressure for quality or benchmarking metrics |
| III. Inner Setting | Structural characteristics (configuration of the inner environment and other tangible materials)  Communications (formal and informal relationships, networks, information sharing practices)  Culture (shared values, beliefs, and norms)  Compatibility and relative priority (fitting within the current workflow, system, and process)  Incentive system (tangible and intangible incentives and rewards and/or disincentives and punishments)  Mission alignment (in line with the overarching goal of the HIV program)  Resources (available resources to implement and deliver) |
| IV. Characteristics of Individuals | High-level leaders (decision-makers, executive leaders, directors)  Mid-level leaders (leaders supervised by a high-level leader and who supervise others)  Opinion leaders (individuals with informal influence on the attitudes and behaviors of others)  Implementors (individuals with subject expertise, individuals leading implementation efforts)  Deliverers and Recipients (individuals who directly or indirectly deliver or receive the services) |
| V. Process of Implementation | Teaming (the degree to which individuals team up, coordinate and collaborate on tasks)  Needs assessment (priorities, preferences, and needs of people)  Context assessment (collect information on current barriers and facilitators)  Planning (identification of roles and responsibilities, outlining specific steps and milestones, defining goals and measures)  Tailoring strategies (choosing and operationalization of implementation activities to address barriers, leverage facilitators and fit context)  Engaging (attract and encourage participation of deliverers and recipients)  Reflecting and Evaluating (collect and discuss quantitative and qualitative information about the success of implementation) |
